# Perceptions and Barriers to Surgical Training of Undergraduates: A Cross-Sectional Survey

**DOI:** 10.7759/cureus.97785

**Published:** 2025-11-25

**Authors:** Christopher P Leiberman, Georgios Kizis

**Affiliations:** 1 Urology, University Hospital Monklands, Airdrie, GBR; 2 Cardiology, University Hospital Wishaw, Wishaw, GBR

**Keywords:** barriers, career, gender disparity, medical students, mentorship, surgical education, work–life balance

## Abstract

Background

Globally, interest in pursuing a surgical career among medical students has declined, largely due to concerns related to lifestyle, competitiveness, and inclusivity. Understanding these early perceptions is essential for maintaining a diverse and adequately staffed surgical workforce. The aim of this study was to identify perceived barriers to pursuing surgical training among third-year undergraduate medical students who had no prior surgical experience.

Methods

A cross-sectional survey was conducted at the University of Glasgow Medical School between February and March 2022. Students were invited via email to complete an anonymous online questionnaire containing a single open-ended question exploring perceived barriers to surgical training. The responses were analysed thematically.

Results

Seventy-eight students responded (77% response rate), providing 85 coded comments. The most frequent barriers were lack of exposure or experience (20%), poor work-life balance (16%), competitiveness of training (15%), and concerns regarding manual dexterity (11%).

Conclusions

Students identified modifiable barriers centred on early exposure, perceptions of lifestyle, and inclusivity. Targeted educational strategies and early mentorship could improve perceptions and support recruitment into surgery.

## Introduction

The decision to pursue a surgical career is shaped by a combination of personal interest, educational exposure, and perceived lifestyle demands. Over the past two decades, studies from both Western and Eastern contexts have reported a decline in interest in surgical careers, often attributed to concerns about work-life balance, training duration, and cultural barriers within the specialty [1-3]. UK data show similar trends, with persistent gender and socioeconomic disparities in surgical training applications [4,5].

Over the past two decades, multiple studies have highlighted concerns regarding medical students' interest in surgery as a career, citing concerns about work-life balance, training duration, and cultural barriers within the specialty [2,4,6,7]. Lifestyle perceptions, especially with regard to long hours, limited flexibility, and competitive training, are consistently associated with lower enthusiasm among medical students [1,8,9].

These perceptions are often compounded by the timing and quality of undergraduate surgical exposure. Without early, positive encounters with the surgical environment, misconceptions may persist and deter students from considering the field [5]. Limited exposure in pre-clinical years can therefore leave students uncertain or intimidated by the operating theatre [10].

In addition, gender disparity remains a persistent issue. Female students frequently report a lack of female role models, discrimination, and difficulty reconciling surgical careers with family life [11-13]. International studies consistently identify similar broad deterrents: lifestyle, competitiveness, and inclusivity [2,3,7]. Addressing these deterrents is essential not only for maintaining a sustainable surgical workforce but also for ensuring diversity and equity within training programs. Recent data from the British Association of Urological Surgeons demonstrates persistent underrepresentation of women and ethnic minority clinicians at national meetings, despite gradual improvement in recent years [14]. Persistent recruitment gaps can strain service delivery and limit innovation, while the underrepresentation of women and students from non-traditional backgrounds undermines efforts to create an inclusive healthcare system [7,13]. Understanding undergraduate perceptions, therefor,e provides crucial insight for designing interventions that strengthen the future surgical workforce [15].

Given these global trends and limited UK undergraduate data, this study aimed to identify perceived barriers to surgical training among third-year medical students with no prior surgical experience. Unlike previous studies conducted among senior students or within specific surgical subspecialties, this study focuses on early undergraduates before formal clinical exposure, providing insight into baseline perceptions that may shape later career intentions [2,3].

## Materials and methods

This cross-sectional survey was conducted among third-year undergraduate medical students at the University of Glasgow Medical School between February and March 2022. At the time of the study, participants had not yet undertaken their surgical clinical rotation.

Students were invited via email to complete an anonymous online questionnaire hosted on Google Forms. The survey consisted of a single open-ended question: 

“What do you consider to be potential barriers to pursuing a surgical career?”

This single-question format was intentionally chosen to elicit spontaneous reflections without constraining responses to predefined categories, allowing richer qualitative insight. No preceding items or prompts were included to avoid priming effects. Responses were exported into Microsoft Excel and analysed using inductive thematic analysis. Two authors independently coded responses and grouped similar statements into themes, resolving discrepancies by consensus. The frequency of each theme was calculated to provide descriptive context.

A total of 78 students responded, yielding 85 coded statements. Demographic information, such as sex or gender, was not collected, as the study aimed to preserve complete anonymity and focus solely on attitudinal responses. This study used anonymised educational data and therefore did not require formal ethics review under institutional policy.

## Results

A total of 101 students were surveyed. Seventy‑eight students responded, yielding a 77% response rate (78/101) and a total of 85 responses. Themes identified and their frequencies are summarised in Table 1. This high response rate likely reflects strong institutional engagement with the survey and the simplicity of the single-question format, which minimised participant burden.

**Table 1:** Perceived barriers to surgical training among third year medical students (n = 85 responses)

Overarching Category	Theme	Illustrative responses	n (%)
Experience and exposure	Lack of exposure or experience	“I’ve had no experience in surgery—it’s a scary concept for me.”; “I would prefer to gain some exposure before deciding.”	17 (19)
Skills and self-efficacy	Manual dexterity or physical limitations	“My lack of dexterity and a bad back.”; “Shaky hands.”	10 (11)
Training, lifestyle, and cultural perceptions	Poor work–life balance	“The lack of a work–life balance.”; “The antisocial hours mainly.”	14 (16)
	Competitiveness of training	“It’s very competitive and I’m probably not clever enough.”; “High competition ratio.”	13 (15)
	Long working hours or intensity	“Long working hours with high‑intensity concentration.”; “The hours.”	9 (10)
	Length of training	“Time it takes to train.”; “Investment of time and difficult working patterns.”	6 (7)
	Limited patient contact	“Less of a relationship with patients compared to medicine.”; “Lack of continuity of care.”	6 (7)
	Male‑dominated culture / lack of female surgeons	“It’s a very male‑dominated field.”; “Not a pleasant place for women to work.”	6 (7)
	Negative work environment / toxic culture	“Heard stories about a toxic work culture.”; “Attitude of some male surgeons.”	4 (5)
Total			85 (100)

Nearly half of all responses were related to lifestyle or training structure, while one in five cited lack of experience. Three overarching categories emerged: (1) experience and exposure; (2) skills and self‑efficacy; and (3) training and lifestyle perceptions.

These barriers often reflected students’ early stage of training. For example, comments pertaining to lack of exposure commonly described anxiety about unfamiliar environments and uncertainty about the surgical workflow. Work-life balance concerns were framed around perceived long hours rather than direct experience, whereas concerns about manual dexterity reflected self-doubt rather than evidence of poor skill. Such distinctions illustrate that most barriers stem from perception rather than experience.

## Discussion

This study provides a snapshot of how early undergraduates perceive surgical training before formal exposure. Three modifiable domains emerge: limited experience, perceived self‑suitability, and lifestyle or cultural barriers.

Although perceived barriers were prevalent, many students expressed curiosity and motivation to acquire surgical skills, suggesting latent interest rather than disengagement. The technical and competitive nature of surgery was regarded ambivalently, motivating some students while deterring others, depending on their confidence and self-efficacy. This duality highlights the importance of educational strategies that amplify positive drivers while systematically correcting misconceptions and apprehensions about surgical training.

Almost one in five students cited lack of exposure or experience as their main barrier, which is consistent with prior reports [2,4,16]. For instance, students who wrote “I’m unsure if I will be able to handle it as I have no experience” support the need for early simulation exposure. Comments such as “It’s a scary concept for me” or “Not much teaching on surgery, so I know very little about it” justify structured pre-clinical teaching. Structured early exposure through workshops, shadowing, and simulation can increase enthusiasm in students [17-19]. Dedicated student-led surgical interest groups have also been shown to foster engagement, mentorship, and early skill development [20]. Students who experience theatre participation and mentorship early in their course develop greater self‑efficacy and familiarity with the surgical environment [10,21].

Early surgical exposure likely improves perceptions through several mechanisms. It normalises the theatre environment, increases self-efficacy, and provides role-model identification, all factors linked to career motivation in social-cognitive theory [22]. Exposure also allows students to see diverse surgeons balancing professional and personal roles, countering misconceptions about lifestyle and inclusivity.

Ten students mentioned manual dexterity or confidence in practical skills. This aligns with research identifying perceived lack of aptitude as a deterrent [6]. Educational interventions, including low‑fidelity bench models and peer‑led teaching, can improve confidence, challenging the misconception that technical ability is innate [18,23].

Perceived poor work-life balance and long training were cited by nearly one‑third of the respondents. Similar findings appear globally [1,3,9]. Such concerns often arise from a limited understanding of contemporary surgical practice. Transparent discussions about flexibility, teamwork, and evolving training models may mitigate these deterrents. Broader sociocultural influences, including the hidden curriculum and perceived exclusivity of surgical environments, may also reinforce these attitudes [24].

References to a “male-dominated field” and “toxic work culture” emphasize the importance of visible mentorship and inclusive teaching environments. These statements mirror qualitative reports of gendered experiences [11-13]. Mentorship and visibility of female surgeons are essential [7,25]. Structured mentoring programmes have demonstrated improved recruitment and confidence among women in surgery [26]. Recent UK data also suggest that pre-clinical experiences and perceptions may significantly shape long-term specialty choices, reinforcing the value of early engagement initiatives [15].

This study has several limitations. First, demographic information, such as sex or gender, was not collected to maintain participant anonymity. As a result, it was not possible to determine whether perceptions of a “male-dominated culture” or “toxic work environment” were more prevalent among female respondents. Given that gender disparity is a recurring theme within surgical education, future studies incorporating demographic data could offer greater insight into how perceptions differ across groups. Additionally, our findings reflect a single UK medical school cohort and may not be generalisable to other settings.

While barriers were the main focus, several responses reflected underlying interest and curiosity about surgery. Many students described a desire for more exposure and teaching rather than a complete lack of interest, suggesting that perceptions are not fixed but modifiable. The strengths of current perceptions lie in this openness to engagement, whereas the weaknesses stem from misperceptions about lifestyle, competitiveness, and inclusivity that persist in the absence of accurate information or mentorship.

Embedding early, structured exposure within pre-clinical curricula, enhancing visible mentorship, and promoting flexible career narratives may help counter misconceptions and improve recruitment. For example, Bartlett et al. demonstrated that participation in a structured “Surgical Shadowing Scheme” significantly improved student enthusiasm and perceived accessibility of surgery [16]. Similarly, Alkatout et al. reported increased an motivation to learn and confidence for decision-making after participation by medical student in a surgery congress [17]. Student-led surgical societies also play a crucial role by providing accessible peer mentorship, skills workshops, and networking opportunities that sustain interest between formal teaching placements [20].

These findings illustrate how targeted interventions can transform curiosity into sustained interest. Future longitudinal studies could track this cohort through their clinical rotations to examine how these baseline perceptions evolve or are mitigated by direct surgical experience.

## Conclusions

Undergraduate students with no surgical exposure perceive surgery as competitive, technically demanding, and poorly compatible with work-life balance. However, the most common deterrent, lack of exposure, is modifiable. Addressing these barriers is critical to securing a diverse and sustainable surgical workforce. Early, inclusive interventions, such as structured exposure, mentorship, and clear messaging around lifestyle balance, can reshape perceptions, enhance recruitment, and ultimately benefit patient care through a workforce that reflects the diversity of the populations it serves.
